# Temporal Lobe Epilepsy Focus Detection Based on the Correlation Between Brain MR Images and EEG Recordings with a Decision Tree

**DOI:** 10.3390/diagnostics14222509

**Published:** 2024-11-09

**Authors:** Cansel Ficici, Ziya Telatar, Osman Erogul, Onur Kocak

**Affiliations:** 1 Department of Electrical and Electronics Engineering, Ankara University, 06830 Ankara, Turkey; 2 Department of Biomedical Engineering, Başkent University, 06790 Ankara, Turkey; ztelatar@baskent.edu.tr (Z.T.); okocak@baskent.edu.tr (O.K.); 3 Department of Biomedical Engineering, TOBB University of Economics and Technology, 06560 Ankara, Turkey; erogul@etu.edu.tr

**Keywords:** EEG, MRI, decision tree, temporal lobe epilepsy, voxel-based morphometry, epileptic focus

## Abstract

Background/Objectives: In this study, a medical decision support system is presented to assist physicians in epileptic focus detection by correlating MRI and EEG data of temporal lobe epilepsy patients. Methods: By exploiting the asymmetry in the hippocampus in MRI images and using voxel-based morphometry analysis, gray matter reduction in the temporal and limbic lobes is detected, and epileptic focus prediction is realized. In addition, an epileptic focus is also determined by calculating the asymmetry score from EEG channels. Finally, epileptic focus detection was performed by associating MRI and EEG data with a decision tree. Results: The results obtained from the proposed algorithm provide 100% overlap with the physician’s finding on the EEG data. Conclusions: MRI and EEG correlation in epileptic focus detection was improved compared with physicians. The proposed algorithm can be used as a medical decision support system for epilepsy diagnosis, treatment, and surgery planning.

## 1. Introduction

In traditional epilepsy studies, epilepsy detection is usually based on EEG data. In cases where the onset and focus of seizures are difficult to distinguish visually from electroencephalography (EEG) recordings, the diagnosis of epilepsy may vary from physician to physician. In this case, the physician examines the patient’s magnetic resonance imaging (MRI) data to detect morphologic abnormalities and volume loss in the brain regions. However, this may be too subtle to be recognized on MRI. In addition, analyzing MRI and EEG data takes a long time, causing delays in the diagnosis and treatment process. In the literature, there are studies that detect epilepsy from EEG recordings and MR images separately, but there are few studies that use these two neuroimaging methods together and detect the epilepsy focus by correlating them.

Recently, some studies have been presented in the literature to detect epileptic seizures from EEG recordings by different feature extraction and classification approaches based on machine learning [1,2,3,4,5,6,7,8] and deep learning methods [9,10,11,12,13,14,15,16]. Ficici et al. [1] presented a study to discriminate epileptic and non-epileptic seizures from EEG signals using DWT-based feature extraction and the boosted tree method. Their highest accuracy was given as 97.2%. Altunay et al. [2] proposed a study to detect epileptic seizures from EEG signals by the linear prediction error energy (LPEE) approach. They obtained 93.6% success in detecting the presence of seizures. Ficici et al. [3] classified interictal, ictal, and normal state periods of EEG signals using support vector machines (SVMs) with nonlinear and linear features. Zarei and Asl [4] presented a method based on DWT and orthogonal matching pursuit (OMP) techniques to detect epilepsy by SVM. Their classifier yielded an average accuracy of 97%. In Slimen et al.’s [5] study, an automated epilepsy detection method based on dual-tree complex wavelet transform was given. Khan et al. [6] introduced the classification of EEG recordings as epileptic and non-epileptic with discriminant analysis. They reported their classification accuracy as a mean accuracy of 99.6% on a clear dataset. Raghu et al. [7] proposed an approach to detect epilepsy with SVM. Their accuracy rate was given as 96.34%. Richhariya et al. [8] presented a method to discriminate seizure and non-seizure epochs by universum support vector machine (USVM).

Natu et al. [9] presented a deep learning-based model combining CNN and RNN to detect epilepsy seizures by extracting spatial and temporal features in EEG signals. They attained a classification accuracy of 98.5%. Ficici et al. [10] detected ictal epochs using a long short-term memory (LSTM) network fed with EEG sub-band features based on discrete wavelet transform (DWT). They achieved classification accuracies of 86.84% and 96.67% on two different EEG datasets. In addition, they identified epileptic focus with an accuracy of 96.10%. Qiu et al. [11] introduced a study for epileptic seizure detection by using a ResNet-LSTM network on the Bonn University dataset, and they achieved an accuracy of 99.78%. Poorani et al. [12] conducted epilepsy detection by using CNN and LSTM methods on the CHB-MIT database. They obtained a classification accuracy of 94.83%. McCallan et al. [17] presented a review of multi-seizure type classification performance based on the Temple University Hospital Seizure Corpus (TUSZ) dataset for focal and generalized classification and multi-seizure type classification.

Also, MRI-based epilepsy detection studies exist in the literature. Ficici et al. [18] presented a study to localize epileptic focus from MR images by using voxel-based morphometry (VBM). Their TLE focus detection results and seizure lateralization from EEG recordings realized by the expert overlapped at a rate of 91.7%. In addition, they obtained a sensitivity of 100% and 80% for right TLE and left TLE detection, respectively. Fearns et al. [19] presented a study to quantitatively assess the diagnostic yield of Morphometric Analysis Program (MAP) feature maps in patients with un-equivocal localization of the epileptic zone and MRI-negative patients. They reported that MAP was useful in detecting previously overlooked subtle structural lesions. However, in truly MRI-negative patients, the additional diagnostic yield was very limited. Yin et al. [20] conducted voxel-based morphology (VBM) and fractional amplitude of low-frequency fluctuation (fALFF) analyses to compare cortical morphology and spontaneous brain activity between drug-naïve benign childhood epilepsy and healthy controls. Aslam et al. [21] described statistical thresholds for asymmetry analysis of hippocampal volumetry and PET to improve the detection of TLE. They stated that threshold parameters warranted further validation in prospective studies. They reported that clinical EEG findings, combined with seizure semiology, could overcome scalp EEG’s limitations and leaned towards MRI lateralization in specific cases. Jber et al. [22] conducted a VBM analysis-based study to find morphometric GM changes in TLE patients. They detected epileptogenic focus by using hippocampal volume. They stated that more subcortical volume and cortical thickness alterations were observed in the left TLE patients than in the right TLE patients by comparison with the healthy control cases. Li et al. [23] proposed a study to evaluate GM abnormalities in MTLE patients. They applied corrected VBM and Surfaced-Based Morphometry (SBM) analysis methods to MR images. They reported that ipsilateral hippocampal atrophy was a characteristic morphological biomarker of MTLE. Shigemoto et al. [24] used a graph theory-based GM network analysis to identify TLE patients. They reported that in TLE groups, GM volume reduction was observed mainly in the ipsilateral hippocampus. Chen et al. [25] detected pediatric MTLE by using the SVM method. They conducted VBM analysis to extract features from MR images. Riederer et al. [26] used the VBM analysis method to detect hippocampal atrophy and seizure focus of MTLE patients. They also applied high-dimensional warping and nonparametric statistics methods. Uher et al. [27] presented a review study that described the recent state-of-the-art computational approaches for image analysis and their potential for epileptogenic zone detection. Also, they reported the current limitations of the methods and possible future directions to augment epileptogenic zone detection. Gupta et al. [28] proposed a study to test the epilepsy prediction accuracy of individual MRI-based tissue types and a novel composite ratio parameter [(GM + WM)/CSF]. They estimated the prediction accuracy of individual MRI tissue types and the composite ratio parameter using EEG results alone or EEG results plus seizure semiology. They reported that the composite ratio provides the highest accuracy of 85% when confirmed by EEG results, while accuracy improved further to 88% when EEG findings and seizure semiology were combined.

On the other hand, there are few studies in the literature in which MRI and EEG findings were compared or evaluated together for the detection of epilepsy. Fallahi et al. [29] presented a decision-making approach to evaluate and compare the performance of neuroimaging markers with clinical findings for the lateralization of mesial temporal lobe epilepsy (mTLE). They used descriptions of seizure semiology, ictal EEG, the ictal epileptogenic zone, the interictal–irritative zone, and MRI findings as clinical features in the Self-Organizing Map (SOM) clustering method. They also used quantitative measurements based on MRI images of T1, FLAIR, and DTI in classification by the SOM method. They used clinical findings and neuroimaging marker features separately. Then, they compared the classification accuracies of neuroimaging markers and clinical findings. Their results showed that the SOM results based on the clinical features could identify all the cases correctly, while the neuroimaging markers made a correct identification at a rate of 94%. Jing et al. [30] examined the relationship between nonlinear analysis of EEG signals, MRI, and single-photon emission computed tomography (SPECT) analyses in epilepsy lateralization of TLE patients. According to the study, they concluded that lateralization by EEG analysis and lateralization by MRI analysis were compatible at 73.9%, EEG analysis and SPECT analysis were compatible at 78.2%, and MRI and SPECT analysis were compatible at 65.2%. 

In this study, an automated decision support system is presented to guide the physician in determining the epileptic focus in TLE patients. In the proposed algorithm, a decision tree method is used based on the correlation of the findings of EEG recordings and MRI images. The analysis of EEG and MRI data was based on the symmetrical nature of the brain. Gray matter reduction and asymmetry of hippocampus volumes were calculated in MRI images. Also, the asymmetry in the energies of symmetric channels was calculated from EEG recordings. The found parameters provide information about the epileptic focus. The experimental results show that the proposed decision tree algorithm achieves 100% success in finding the epileptic focus of TLE patients compared with the physician’s findings.

This article is organized as follows: Section 2 introduces the EEG and MRI datasets used in this study. Furthermore, the algorithm is explained in detail using flowcharts and theoretical backgrounds. In Section 3, MRI and EEG analysis results are reported. The novelty of this paper and contributions and conclusions are presented in Section 4.

## 2. Materials and Methods

### 2.1. Description of the Dataset and Framework of This Study

In this study, multi-channel EEG recordings and brain MR images of TLE patients obtained from the Ankara University hospital were used. EEG signals with a sampling frequency of 500 Hz were received from 18 channels according to a 10–20 electrode placement system. EEG signals were recorded from Fp1-F7, F7-T3, T3-T5, T5-O1, Fp2-F8, F8-T4, T4-T6, T6-O2, Fp1-F3, F3-C3, C3-P3, P3-O1, Fp2-F4, F4-C4, C4-P4, P4-O2, FZ-CZ, and CZ-PZ channels. The EEG database used in this study includes 76 seizure recordings (48 right TLE, 28 left TLE) of 26 TLE patients. Epileptic seizure and seizure intervals were labeled on EEG signals by the expert retrospectively. Rhythmic theta and beta activities, repetitive spikes, sharp waves, and low-voltage rapid activity were the epileptic seizure indicators for the expert. Also, left TLE and right TLE patients were labeled by the expert based on the findings of EEG recordings and seizure semiology. The MRI dataset used in this study was obtained from 15 TLE patients diagnosed with MTS, HS, hippocampal atrophy, or MRI-negative, and 14 control subjects. Hippocampus areas were labeled on MR images by the expert. Atrophy observed on the hippocampus area on coronal T1-weighted MRI, increased T2 signal reflecting gliosis, and abnormal hippocampus morphology on T1-weighted images were the indicators of hippocampal sclerosis diagnosis for the expert. Expert labeling on EEG recordings and MR images was considered the gold standard for the performance evaluation of this study.

**Figure 1:**
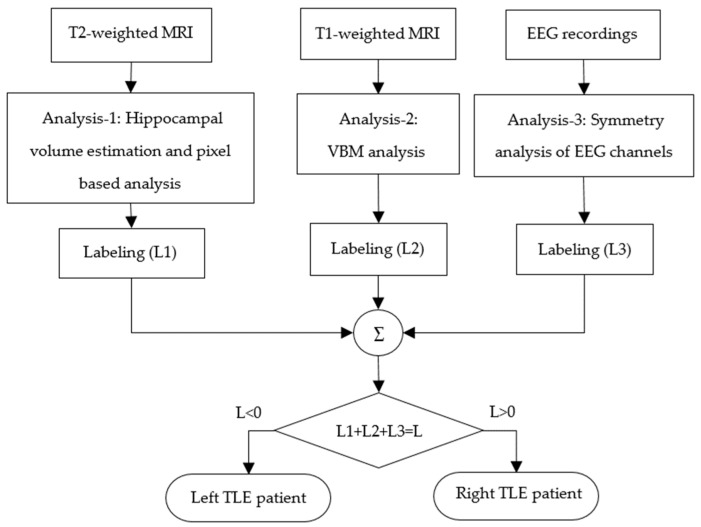
Flowchart of TLE detection based on correlation of EEG and MRI.

This proposed study was implemented with Matlab 2021a [31] via a computer, Intel Core i7, 2.60 GHz processor, with 16.0 GB RAM. A flowchart of the proposed approach for TLE detection based on the correlation between EEG and MRI is given in Figure 1 [32]. Firstly, in the hippocampal volume estimation and pixel-based analysis part, the mean and standard deviation of the gray-level pixel intensities of the left and right hippocampus areas on T2-weighted MR images were calculated. In addition, gray-level MR images were masked with the hippocampus regions labeled by the expert and then converted into binary images. After that, the left and right hippocampus volumes were automatically calculated by the algorithm. Left and right hippocampus volume, mean value of pixel intensities, and standard deviation features were used to label MR images as left TLE and right TLE by using machine learning. Secondly, in the VBM analysis part, T1-weighted MR images were used to determine whether TLE patients had GM reduction in the temporal lobe or limbic lobe regions. If GM reduction was detected, the patient was labeled as either left TLE or right TLE based on the cerebral hemisphere where GM reduction was found. Thirdly, in the symmetry analysis of EEG channels part, energies of ictal EEG signals were calculated for each channel. Then, the asymmetry scores were calculated from the left and right symmetric EEG channels. According to the asymmetry score, seizure recordings were labeled as either left TLE or right TLE. In the decision tree, L1, L2, and L3 represent the lateralization labels of Analysis-1 (hippocampal volume estimation and pixel-based analysis), Analysis-2 (VBM analysis), and Analysis-3 (symmetry analysis of EEG channels). The number “−1” was assigned to the left TLE class, the number “1” to the right TLE class, and the number “0” to MR-negative. Finally, the epileptic focus was determined by correlating the decisions obtained from MR images and EEG recordings by using a decision tree. In the decision tree, label numbers assigned in Analysis-1, Analysis-2, and Analysis-3 were summed. If the total number was greater than zero, the epileptic focus for that patient was ultimately determined to be on the left brain hemisphere; otherwise, the epileptic focus was on the right hemisphere.

### 2.2. Hippocampal Volume Estimation and Pixel-Based Analysis of MR Images

**Figure 2:**
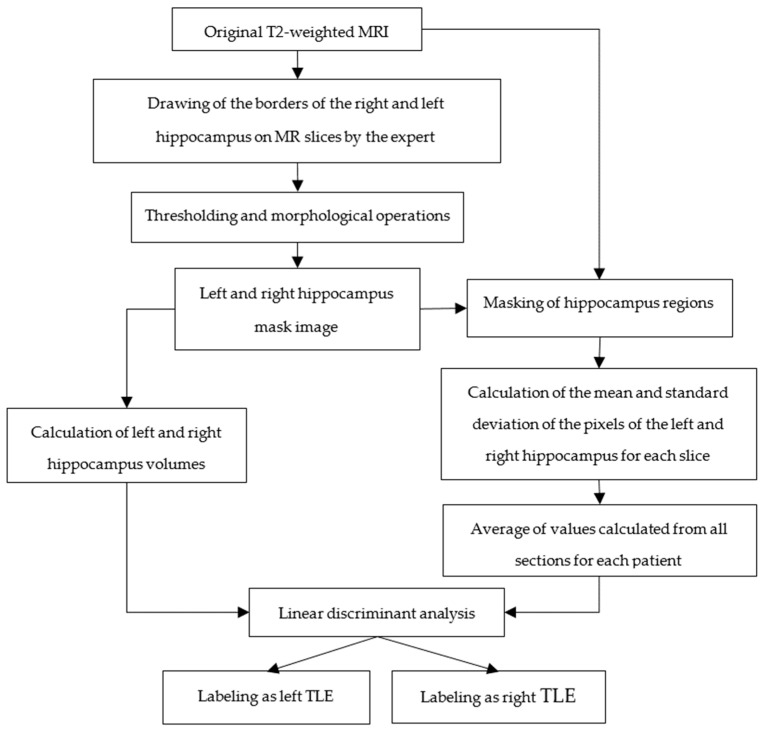
Flowchart of TLE localization from the hippocampus on MRI.

Abnormal conditions such as atrophy and gliosis in the hippocampus of TLE patients cause an increase in signal intensity and a decrease in hippocampus volumes on T2-weighted MR images. Therefore, in this part of this study, epileptic focus detection from subtle signal increases in T2-weighted images and volume reductions in hippocampus regions were realized. A flowchart of the TLE localization from hippocampus on MRI is given in Figure 2 [32].

**Figure 3:**
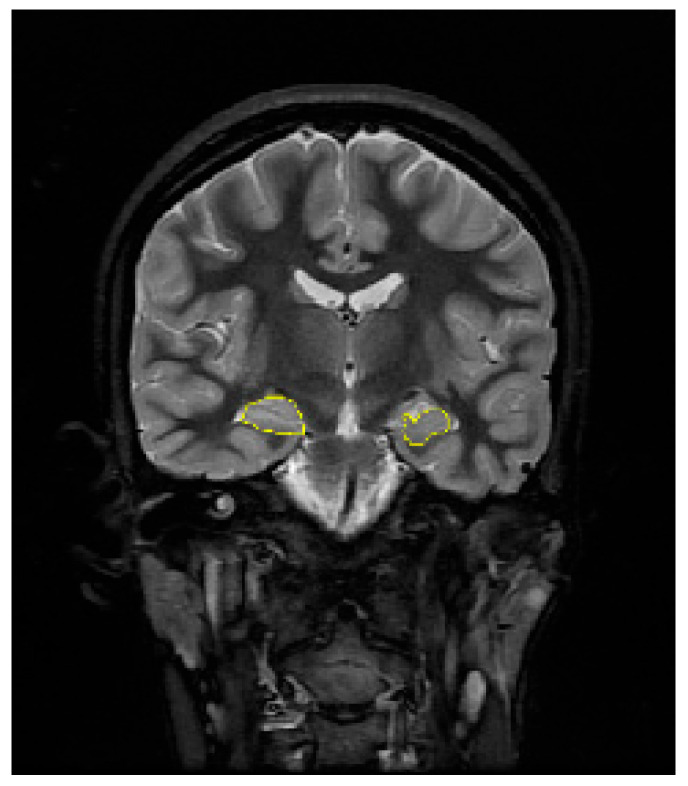
MRI slice of a TLE patient with hippocampus boundaries drawn in yellow by an expert.

First of all, hippocampus boundaries were drawn by the expert in T2-weighted coronal MR images of TLE patients. A sample of a T2-weighted coronal MR slice of a TLE patient with hippocampus boundaries drawn by the expert is shown in Figure 3. Then, binary hippocampus mask images were obtained by thresholding the images labeled by the expert. Left and right hippocampus volumes were calculated from these binary hippocampus images. In addition, the original T2-weighted MR images were masked with the binary hippocampus images. The mean and standard deviation of the pixel intensities of the left and right hippocampus regions were calculated for each gray level MR slice. Volume estimation from hippocampus mask images was realized with (1) and (2). The mean and standard deviation of the pixel intensities of the left and right hippocampus were calculated for each section by using (3) and (4), respectively. The values calculated from all slices of each patient were averaged. These features were used in the linear discriminant analysis (LDA) method; thus, left TLE and right TLE classification was conducted.

(1) Area=(number of pixels) x (pixel area),

(2) $$Volume=\sum_{i}{Area}_{i}\;\;x\;slice\;thickness,$$

(3) $$\mu =\frac{1}{N}\sum_{n=1}^{N}x\left(n\right),$$

(4) $$\sigma =\sqrt{\frac{1}{N-1}\sum_{n=1}^{N}{\left(x\left(n\right)-\mu \right)}^{2}}.$$

### 2.3. VBM Analysis from MR Images

**Figure 4:**
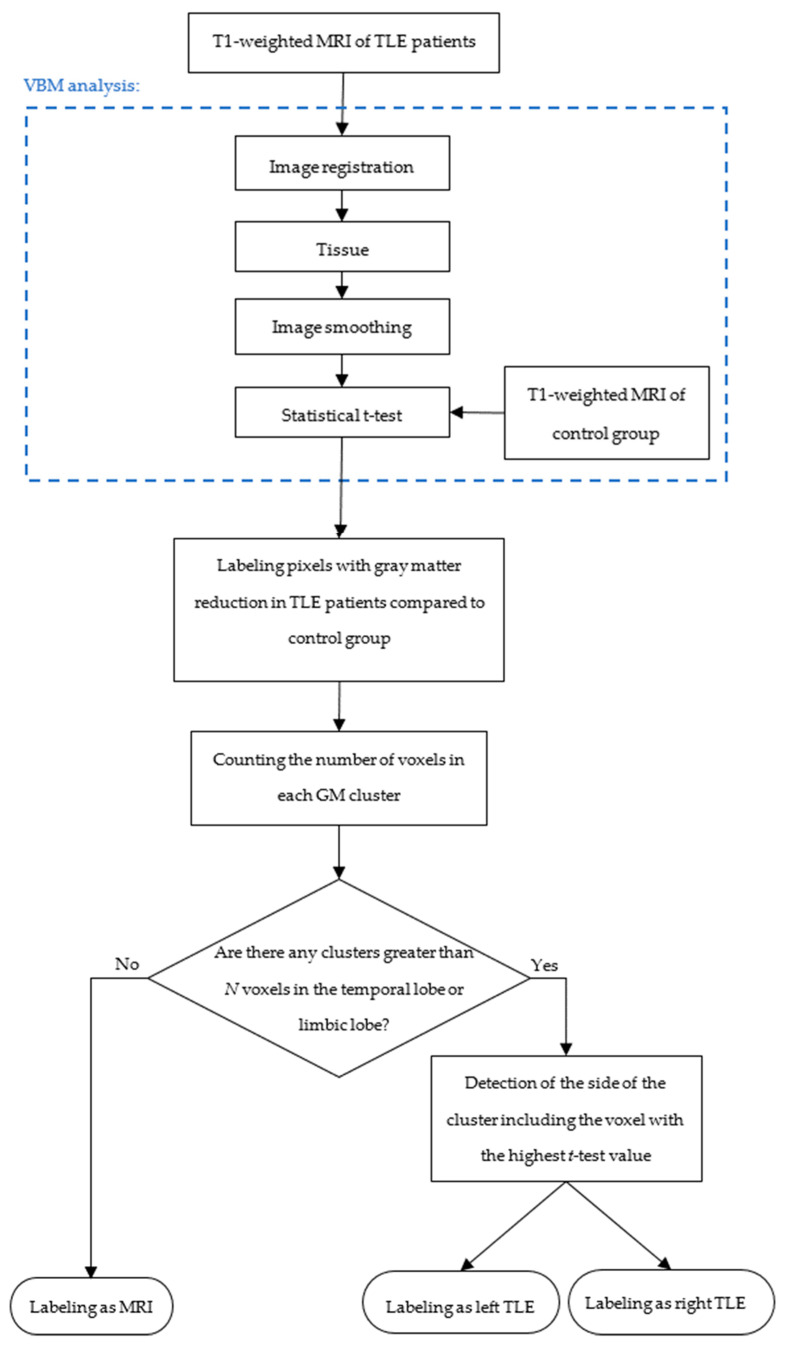
Flowchart of TLE localization using VBM analysis applied on MRI.

In TLE patients, GM reduction generally occurs in the hippocampus and structures of the limbic system and mesial temporal lobe [33]. So, the region of interest (ROI) of VBM analysis in this study is the limbic lobe and temporal lobe. By applying the VBM method, GM reduction areas were detected from the T1-weighted MR images of TLE patients by comparing them with the T1-weighted MR images of the control group [18]. A flowchart of the TLE localization using VBM analysis is given in Figure 4. The region with GM reduction is labeled as the epileptic focus. If no GM reduction was found in the temporal lobe or limbic lobe, that patient was labeled as MR-negative.

VBM analysis is based on the statistical *t*-test. The decrease in GM in MRI images of epilepsy patients compared to the control group was calculated using the *t*-test.

The first step of VBM analysis is image registration enabling the overlapping of boundaries of MR images taken from different databases [34]. The second step is normalization and then smoothing GM images with a Gaussian filter. The final step of the VBM method is the segmentation of white matter, gray matter, and cerebrospinal fluid parts in MR images by applying the unified segmentation based on the Gaussian Mixture Model (GMM) [35]. Threshold values at voxel and cluster levels were determined experimentally. In the *t*-test analysis in this study, threshold values of *p* < 0.001 or *p* < 0.003 were considered significantly different to detect GM changes in each patient with TLE. Furthermore, a cluster size greater than 50 voxels (N value given in Figure 3) was considered an appropriate threshold value to determine the epilepsy focus [32].

**Figure 5:**
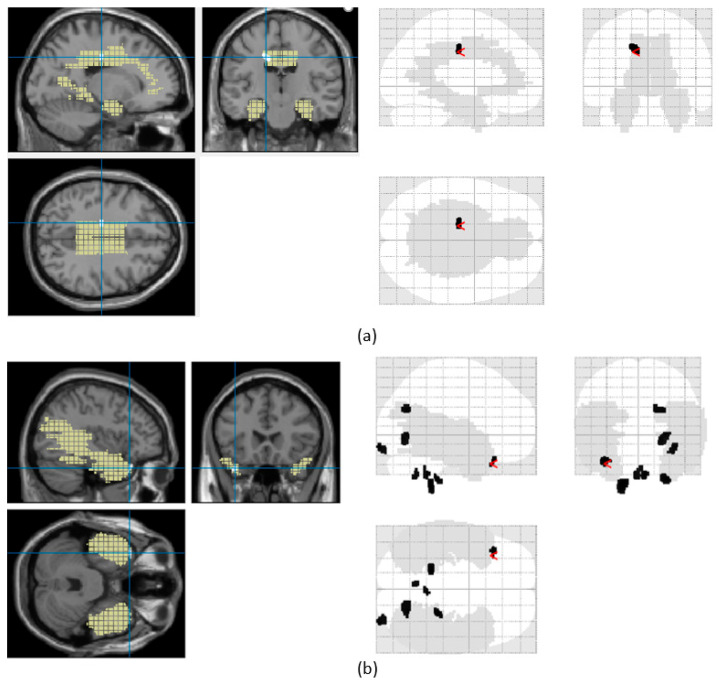
Area with significant gray matter reduction in the (**a**) limbic lobe area and the (**b**) temporal lobe area detected by VBM analysis for two patients from the AU dataset.

An area with significant gray matter reduction in the limbic lobe and the temporal lobe area detected by VBM analysis for two patients from the AU dataset are shown in Figure 5a and Figure 5b, respectively. Figure 5 shows the glass views of the MR slices (left column) and the corresponding gray-level images (right column). In the glass view images, voxels in GM reduction areas are indicated by black-shaded areas, while the gray-shaded areas are specific regions selected by the user. In this study, temporal lobe and limbic lobe areas were selected for TLE analysis. In the gray-level images, GM atrophy is shown with white shaded areas, while temporal lobe and limbic lobe areas are shown in yellow. Once the highest *t*-test value was determined, the cluster containing this voxel was found and marked with a red arrow (Figure 5).

### 2.4. Symmetry Analysis of EEG Signals

**Figure 6:**
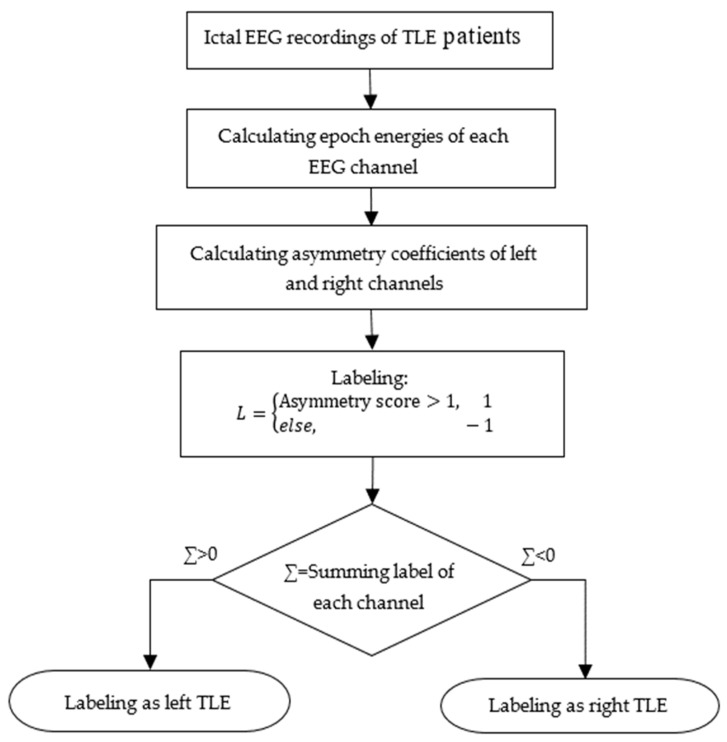
Flowchart of TLE localization by using symmetry analysis of EEG signal channels.

In this part, ictal EEG signal energies were calculated for each left and right symmetric EEG channel; then, the asymmetry score was calculated. A flowchart of TLE localization using symmetry analysis of EEG signal channels is given in Figure 6 [32]. The asymmetry score was calculated from the energy ratios of these symmetrical left and right bipolar EEG channels. Symmetrical EEG channel pairs are given in Table 1. The asymmetry score represented by A was calculated by using (5) for each channel [10]. $x_{left}$ and $x_{right}$ represent symmetric left and right EEG channel signals for epoch length, respectively.

(5) $$A=\frac{\sum {x_{left}(n)}^{2}}{\sum {x_{right}(n)}^{2}}$$

**Figure 7:**
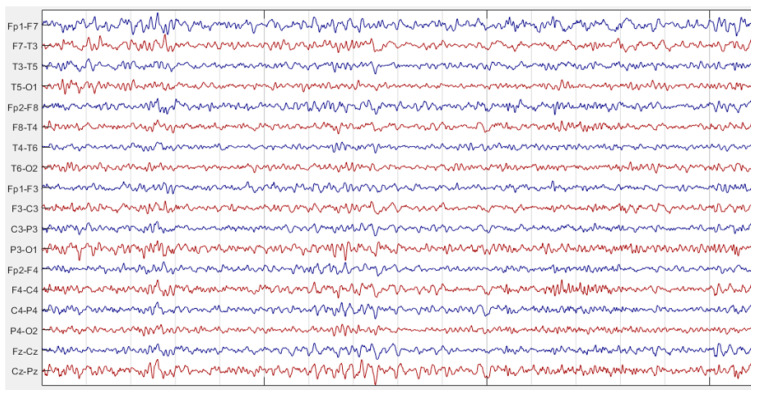
EEG recording of a patient in a seizure-free interval.

**Figure 8:**
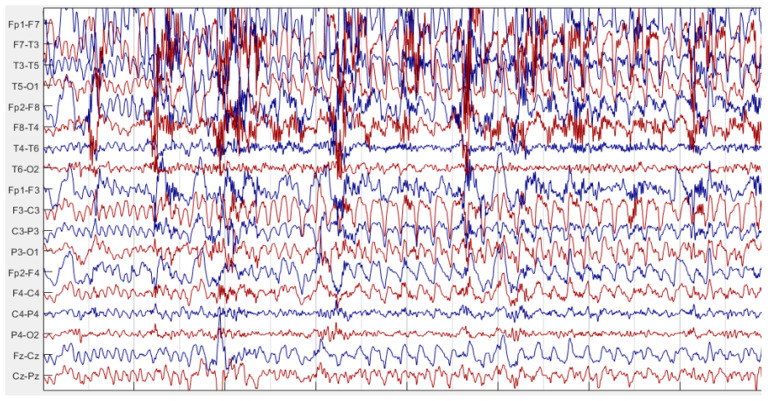
EEG recording of a patient during an epileptic seizure.

Examples of EEG recordings in seizure-free and epileptic seizure intervals are given in Figure 7 and Figure 8, respectively. These figures were obtained by using Matlab [36]. By comparing Figure 7 and Figure 8, it can be seen that excessive neural activity during the seizure causes an increase in EEG signal energy on the epileptic focus side. So, if the asymmetry score was greater than 1, EEG channel pair of the patient was labeled as “1”; otherwise, it was labeled as “−1”. Then, the label numbers were summed. If the total number was greater than zero, that recording was labeled as left sided focus; otherwise; it was labeled as right sided focus. This labeling was realized for each seizure recording of each patient. If the seizure recordings labeled as the left focus exceed the number of seizure recordings labeled as the right focus, that patient was determined as a left TLE patient; otherwise, the patient was labeled as a right TLE patient.

## 3. Results

In this study, MRI and EEG data were analyzed, and results were obtained using the decision tree algorithm. For EEG data, the energies of symmetric EEG channels were calculated, and asymmetry coefficients were calculated and labeled as left or right epileptic focus. In addition, separate epileptic focus detection analyses were performed with T2- and T1-weighted MR images, and as a result, left and right foci were labeled. Afterward, a final decision was determined for the epileptic focus with the decision tree algorithm.

The results for Analysis-1 given in Figure 1 and Figure 2 are presented in Table 2, Table 3 and Table 4. Left and right hippocampus volumes, statistical features of hippocampal regions, which are the mean value and standard deviation of pixels in the left and right hippocampus regions, were used as feature vectors in different machine learning methods (decision tree, discriminant analysis, Naïve Bayes, SVM, k-nearest neighbor, boosting, bagging, random subsampling, ANN) with different combinations for TLE classification. The results are given in Table 2, Table 3 and Table 4. The results of Analysis-1 using hippocampus volumes and statistical features of hippocampal regions for two-class classification (TLE and control group), three-class classification (LTLE, RTLE, and control group), and another two-class classification (LTLE and RTLE) are given in Table 2, Table 3, and Table 4, respectively. As can be seen from these tables, the common feature with the highest classification accuracy across all classification groups is the left and right hippocampus volumes.

In addition, the LDA method produced the highest accuracy, sensitivity, and PPV (Positive Prediction Value) by handling left and right hippocampus volume features.

The results for Analysis-2 given in Figure 1 and Figure 4 are presented in Table 5 [18]. In Table 5, the location of the highest *t*-test value in the limbic lobe and temporal lobe areas detected by using VBM analysis are given. Also, cluster size, *t*-test values, and MNI coordinates are given. According to the location of the peak voxel, the TLE focus side is determined as the left or right hemisphere. Since the absence of an epileptogenic lesion or atrophy on visual inspection of the MRI conducted by the expert was defined as MRI-negative TLE, the absence of the GM reduction in the temporal lobe or the limbic lobe was defined as MRI-negative in the VBM analysis.

The physician’s results of epileptic focus detection separately from EEG and MRI are compared with the results of Analysis-2. It was seen that the VBM method gives more consistent results with the expert decision from EEG (accuracy of 91.7%) than the physician’s evaluation for separate EEG and MRI data (accuracy of 50%).

The results for Analysis-3 shown in Figure 1 and Figure 6 are presented in Table 6 [10]. As seen in Table 6, the accuracy, sensitivity, and specificity values for left and right epileptic focus detection from EEG signals are 96.1%, 100%, and 93.8%, respectively.

The MRI and EEG lateralization of the physician and the proposed method are given in Table 7. Since the MRI of the first patient in this table consists of images taken after temporal lobectomy, the hippocampus volume calculation could not be performed, so Analysis-1 could not be applied to this patient.

The results of the method of correlating MRI and EEG data are shown in Table 8. This table indicates the following:

- The physician detected epilepsy findings (HS, hippocampal atrophy, mesial temporal sclerosis) in the MR images of 6 out of the 15 TLE patients. The MRI lateralization of patients with epilepsy detected by the physician on MRI images and the physician’s EEG lateralization in these patients overlap by 50% (3/6).
- The physician’s lateralization of epilepsy from EEG recordings and the MRI lateralization of Analysis-1 overlap by 78.6% (11/14).
- Analysis-2 detected epilepsy on MRI in 80.0% (12/15) of the TLE patients. The MRI lateralization of patients with epilepsy detected by the VBM algorithm (Analysis-2) and the lateralization made by the physician from EEG data overlap by 91.7% (11/12).
- The epilepsy lateralization made by the physician from the EEG recordings and the EEG lateralization of Analysis-3 overlap by 86.7% (13/15).
- The lateralization of epilepsy made by the physician from EEG recordings and the lateralization of the proposed MRI and EEG data correlation algorithm (decision tree) overlap by 100% (15/15).
- The MRI lateralization of patients with epilepsy detected by the physician on MRI images and the MRI lateralization of Analysis-1 overlap by 60% (3/5) (when calculating this overlap rate, patient 1 with temporal lobectomy was not included).
- The MRI lateralization of patients with gray matter reduction detected by the VBM analysis (Analysis-2) and the EEG lateralization of Analysis-3 in these patients overlap by 91.7% (11/12).
- The proposed VBM analysis algorithm (Analysis-2) detected epilepsy in 80.0% (12/15) of the TLE patients, while the physician detected epilepsy in the MR images of 40% (6/15) of the TLE patients.

The overlap ratio of the proposed MRI and EEG correlation algorithm (decision tree) with the proposed EEG lateralization algorithm (Analysis-3) is 100% in patients with epilepsy. The overlap rate of EEG and MRI lateralization by the physician in patients where the MRI findings are detected is 50%. Therefore, both MRI finding detection and MRI and EEG lateralization overlap rates were improved compared with the physician.

Table 9 shows a comparison of the epileptic focus detection studies available in the literature with the proposed method. To the best of our knowledge, there are few automatic epileptic focus detection studies in the literature that use MRI and EEG data together. In the studies given in Table 9, epileptic focus detections were performed on separate MRI or EEG data and on MRI and EEG data together. There is a 100% match between the proposed algorithm and the physician evaluation results. When compared with the EEG and MRI studies in the literature, the highest accuracy in the detection of epileptic focus is achieved by the proposed algorithm.

## 4. Discussion and Conclusions

In this study, by correlating MRI and EEG data of TLE patients, an automated decision support system is obtained to assist physicians in epileptic focus detection. In this respect, EEG signals and MRI images of TLE patients were analyzed, and epilepsy disease was detected with the decision tree algorithm.

T1- and T2-weighted images were used in the analysis of MR images. By performing VBM analysis using T1-weighted images, areas with gray matter reduction in the brains of epilepsy patients were detected. Using T2-weighted images, the hippocampus volumes of epilepsy patients and the asymmetry parameters of the right and left hippocampus were calculated. Thus, it was decided whether there was an asymmetry between the left and right hippocampus by using the LDA method. In addition, the asymmetry coefficient was obtained from ictal EEG signals recorded through symmetrical EEG channels in the left and right brain lobes. In the last stage, labels were assigned to the results obtained from the analysis of MRI images and EEG signals. These labels formed the inputs of the decision tree algorithm. The output of the algorithm made the final decision of epileptic focus.

There are many studies on epilepsy detection from EEG signals alone in the literature. In addition, there are also studies that applied VBM analysis to MR images alone. The novelty of this study is that it utilizes the correlation in the information provided by both neuroimaging techniques as a decision tree for epilepsy detection.

Our study outperforms other related studies because of its comprehensive approach to detecting TLE by combining multiple advanced neuroimaging and electrophysiological features that capture unique aspects of the disorder. Specifically, our study integrates precise gray matter reductions identified in both the limbic and temporal lobes, advanced statistical analysis of hippocampal pixel properties in MR images, and asymmetry measures of both hippocampal volumes and EEG signal energies across channels. This multifaceted approach enhances the accuracy of TLE detection by leveraging complementary insights from structural, functional, and statistical data—something most related studies may not achieve when focusing on a limited set of features. Consequently, this comprehensive feature set enables our model to more effectively capture TLE-related abnormalities, leading to better diagnostic performance and potentially higher clinical relevance.

The main contributions of this study are as follows: (1) It was shown that the detection of epileptic focus by correlation of EEG and MR images provides higher success compared to the evaluation of EEG and MRI analysis separately in the detection of epileptic focus. (2) VBM analysis enabled the detection of GM reductions in MR images of epilepsy patients that cannot be visually detected by the physician. (3) Unique distinguishing features were obtained in the detection of TLE patients. These features are GM reductions detected in the limbic lobe and temporal lobe, statistical properties of pixels belonging to hippocampus regions in MR images, asymmetry in hippocampus volumes, and asymmetry in EEG signal channel energies.

## Figures and Tables

**Table 1:** Symmetrical EEG channels.

	Left Side	Right side
1	Fp1-F7	Fp2-F8
2	F7-T3	F8-T4
3	T3-T5	T4-T6
4	T5-O1	T6-O2
5	Fp1-F3	Fp2-F4
6	F3-C3	F4-C4
7	C3-P3	C4-P4
8	P3-O1	P4-O2

**Table 2:** Results of Analysis-1 using hippocampus volumes and statistical features of hippocampal regions in TLE and control group classification.

Classes	Feature Combinations	Method	Accuracy (%)	Sensitivity (%)	PPV (%)
14 TLE, 14 control group	mean	Naïve Bayes	78.6	85.7	75.0
	standard deviation	KNN	82.1	92.9	76.5
	mean and standard deviation	Decision tree	82.1	85.7	80.0
	mean and standard deviation and hippocampus volumes	KNN	85.7	92.9	83.1
	hippocampus volumes	Naïve Bayes	89.3	92.9	86.7
	mean and hippocampus volumes	Naïve Bayes	89.3	92.9	86.7
	standard deviation and hippocampus volumes	KNN	82.1	92.9	76.5

**Table 3:** Results of Analysis-1 using hippocampus volumes and statistical features of hippocampal regions in LTLE, RTLE, and control group classification.

Classes	Feature Combinations	Method	Accuracy (%)	Sensitivity (%)	PPV (%)
six LTLE, eight RTLE, 14 control group	mean	LDA	67.9	58.9	74.1
	standard deviation	Naïve Bayes	57.1	47.2	43.2
	mean and standard deviation	ANN	57.1	45.8	47.2
	mean and standard deviation and hippocampus volumes	KNN	78.6	73.9	76.0
	hippocampus volumes	LDA	78.6	74.0	77.1
	mean and hippocampus volumes	Naïve Bayes	71.4	60.6	60.3
	standard deviation and hippocampus volumes	LDA	67.9	60.8	62.1

**Table 4:** Results of Analysis-1 using hippocampus volumes and statistical features of hippocampal regions in LTLE and RTLE classification.

Classes	Feature Combinations	Method	Accuracy (%)	Sensitivity (%)	PPV (%)
six LTLE, eight RTLE	mean	SVM	64.3	83.3	55.6
	standard deviation	Naïve Bayes	57.1	50.0	50.0
	mean and standard deviation	SVM	57.1	0	-
	mean and standard deviation and hippocampus volumes	LDA	71.4	66.7	75.0
	hippocampus volumes	LDA	78.6	66.7	80.0
	mean and hippocampus volumes	KNN	71.4	50.0	75.0
	standard deviation and hippocampus volumes	ANN	78.6	66.7	80.0

**Table 5:** Detection of the highest *t*-test value and its region by using VBM analysis.

Patient No.	Location of Peak Voxels	Cluster Size (Number of Voxels)	Voxel *t* Statistic (Peak Voxel)	MNI Coordinates (x, y, z)	TLE Focus Side Decision
1	//Right Cerebrum//Limbic Lobe//Right Hippocampus	19837	13.07	30, −20, −15	Right
2	//Left Cerebrum//Temporal Lobe//Temporal Gyrus	51	4.56	−38, 24, −28	Left
3	//Left Cerebrum//Limbic Lobe//Cingulate Gyrus	74	4.3026	−16.5, −18, 37.5	Left
4	NaN*	NaN	NaN	NaN	MRI-negative
5	//Right Cerebrum//Limbic Lobe//Right Hippocampus	172	6.53	32, −12, −20	Right
6	//Right Cerebrum//Temporal Lobe//Middle Temporal Gyrus	83	5.0754	48, −36, 0	Right
7	//Left Cerebrum//Limbic Lobe//Left Hippocampus	337	4.63	−33, −14, −20	Left
8	//Left Cerebrum//Temporal Lobe//Temporal Gyrus	4096	11.71	−40, 8, −26	Left
9	//Right Cerebrum//Temporal Lobe//Right Hippocampus	108	4.7375	−27, −39, 0	Right
10	//Right Cerebrum//Limbic Lobe//Right Hippocampus	54	4.6149	31.5, −12, −19.5	Right
11	//Left Cerebrum//Temporal Lobe//Left Middle Temporal Gyrus	14862	16.10	−38, 20, −36	Left
12	//Left Cerebrum//Limbic Lobe//Parahippocampal Gyrus	50	4.5347	−28.5, −58.5, −7.5	Left
13	NaN	NaN	NaN	NaN	MRI-negative
14	NaN	NaN	NaN	NaN	MRI-negative
15	//Left Cerebrum//Limbic Lobe//Left Anterior Cingulate and Paracingulate Gyri	61	4.88	−4, 48, 2	Left

NaN* indicates that GM reduction was not detected in the temporal lobe or in the limbic lobe.

**Table 6:** Results of symmetry analysis of the EEG signal algorithm.

Number of EEG Data	Result
76 (48 RTLE, 28 LTLE)	Accuracy = 96.1%
	Sensitivity = 100%
	Specificity = 93.8%

**Table 7:** Expert findings and decisions of the proposed method on TLE diagnosis.

TLE Patient No	Expert Findings from EEG	Expert Findings from MRI	Decision of Analysis-1	Decision of Analysis-2	Decision of Analysis-3	Decision Tree Final Result
1	Right TLE	Right Mesial Temporal Sclerosis	NaN*	Right TLE	Right TLE	Right TLE
2	Left TLE	Right Hippocampal Sclerosis	Right TLE	Left TLE	Left TLE	Left TLE
3	Left TLE	MRI-negative	Left TLE	Left TLE	Left TLE	Left TLE
4	Right TLE	MRI-negative	Right TLE	MRI negative	Left TLE	Right TLE
5	Right TLE	MRI-negative	Right TLE	Right TLE	Right TLE	Right TLE
6	Right TLE	MRI-negative	Right TLE	Right TLE	Right TLE	Right TLE
7	Left TLE	MRI-negative	Left TLE	Left TLE	Left TLE	Left TLE
8	Right TLE	Bilateral Hippocampal Atrophy	Right TLE	Left TLE	Right TLE	Right TLE
9	Right TLE	Bilateral Hippocampal Atrophy	Right TLE	Right TLE	Right TLE	Right TLE
10	Right TLE	MRI-negative	Left TLE	Right TLE	Right TLE	Right TLE
11	Left TLE	Left Hippocampal Atrophy	Left TLE	Left TLE	Left TLE	Left TLE
12	Left TLE	MRI-negative	Right TLE	Left TLE	Left TLE	Left TLE
13	Right TLE	Right Hippocampal Atrophy	Right TLE	MRI negative	Left TLE	Right TLE
14	Right TLE	MRI-negative	Right TLE	MRI negative	Right TLE	Right TLE
15	Left TLE	MRI-negative	Left TLE	Left TLE	Left TLE	Left TLE

NaN* indicates that Patient-1 was excluded from hippocampal volume estimation (Analysis-1) due to a history of temporal lobectomy.

**Table 8:** The rates of overlap between expert findings and decisions of the proposed method on TLE.

Gold Standard		Proposed Method				Results
Expert Lateralization from EEG	Expert Lateralization from MRI	Lateralization of Analysis-1	Lateralization of Analysis-2	Lateralization of Analysis-3	Lateralization of the Decision Tree	Lateralization Overlap Ratio
√	√					50%
√		√				78.6%
√			√			91.7%
√				√		86.7%
√					√	100%
	√	√				60%
	√		√			50%
			√	√		91.7%

**Table 9:** Table showing a comparison of the proposed method with existing epileptic focus detection studies.

Authors	Dataset	Algorithm/Method	Results
Chen et al. [25]	Shenzhen Children’s Hospital: 22 MTLE-HS patients (16 left, six right) and 15 healthy controls	Right-HS and control group classification by using volumes of regions with GM reduction from MRI/VBM and SVM	Accuracy: 93.3% Sensitivity: 100% Specificity: 90.7%
		Left-HS and control group classification by using volumes of regions with GM reduction from MRI/VBM and SVM	Accuracy: 80.0% Sensitivity: 80.0% Specificity: 80.0%
Behesti et al. [37]	National Center of Neurology and Psychiatry Hospital: 63 participants, including 24 HCs, 19 MRI-negative, PET-positive left TLE patients and 20 MRI-negative, PET-positive right TLE	Discrimination of left TLE, right TLE by using MR images obtained by SPM12 toolbox/SVM	Accuracy: 66.7% Sensitivity: 63.2% Specificity: 70%
Nazem-Zadeh et al. [38]	Dataset source not available in the original manuscript. The authors implemented the study in Henry Ford Hospital: 10 LTLE, 10 RTLE, 45 control group	Classification of left and right TLE patients from MRI by using hippocampus volumes/Hemispheric Variation Uncertainty (HVU) analysis	Accuracy: 84.9%
Jafari-Khouzani et al. [39]	Dataset source not available in the original manuscript. The authors implemented the study in Henry Ford Hospital.: 20 LTLE, 16 RTLE, 25 control group	Classification of left and right TLE patients from MRI by using hippocampus volumes/LDA	Accuracy: 75.3%
Türk et al. [40]	Bonn University: 500 EEG segments with 23.6 s duration for each	Epileptic focus identification from EEG/convolutional neural network (CNN)	Accuracy: 98.5%
Daoud et al. [41]	Bern–Barcelona and Bonn University: 7500 EEG segments with 20 sec duration for each	Epileptic focus identification from EEG/Deep convolutional autoencoder	Accuracy: 96.0%
Fallahi et al. [28]	Tehran University: 35 mesial TLE patients	Presurgical lateralization of mesial temporal lobe epilepsy from EEG and MRI/self-organizing maps	Accuracy: 94.0%
Jing et al. [30]	Dataset source not available in the paper: 23 TLE patient	Investigated the correlation between lateralization MRI, SPECT, and EEG/nonlinear analysis	Concordance between EEG and MRI: 73.9% Concordance between EEG and SPECT: 78.2% Concordance between MRI and SPECT: 65.2%
Proposed study	University Hospital: 15 TLE (six LTLE, nine RTLE)	Epileptic focus detection/decision tree	Accuracy: 100%

## Data Availability

Data are unavailable because of privacy and ethical restrictions.

## References

[1] Ficici C., Telatar Z., Eroğul O. (2022). Automated temporal lobe epilepsy and psychogenic nonepileptic seizure patient discrimination from multichannel EEG recordings using DWT based analysis. Biomed. Signal Process. Control.

[2] Altunay S., Telatar Z., Erogul O. (2010). Epileptic EEG detection using the linear prediction error energy. Expert Syst. Appl..

[3] Ficici C., Erogul O., Telatar Z. Epileptic Activity Detection in EEG Signals using Linear and Non-linear Feature Extraction Methods. Proceedings of the 2019 11th International Conference on Electrical and Electronics Engineering (ELECO).

[4] Zarei A., Asl B.M. (2021). Automatic seizure detection using orthogonal matching pursuit, discrete wavelet transform, and entropy based features of EEG signals. Comput. Biol. Med..

[5] Slimen I.B., Boubchir L., Mbarki Z., Seddik H. (2020). Algorithms, EEG epileptic seizure detection and classification based on dual-tree complex wavelet transform and machine learning. J. Biomed. Res..

[6] Khan K.A., Shanir P.P., Khan Y.U., Farooq O. (2020). A hybrid Local Binary Pattern and wavelets based approach for EEG classification for diagnosing epilepsy. Expert Syst. Appl..

[7] Raghu S., Sriraam N., Temel Y., Rao S.V., Hegde A.S., Kubben P.L. (2019). Performance evaluation of DWT based sigmoid entropy in time and frequency domains for automated detection of epileptic seizures using SVM classifier. Comput. Biol. Med..

[8] Richhariya B., Tanveer M. (2018). EEG signal classification using universum support vector machine. Expert Syst. Appl..

[9] Natu M., Bachute M., Kotecha K. (2023). HCLA_CBiGRU: Hybrid Convolutional Bidirectional GRU Based Model for Epileptic Seizure Detection. Neurosci. J..

[10] Ficici C., Telatar Z., Kocak O., Erogul O. (2023). Identification of TLE Focus from EEG Signals by Using Deep Learning Approach. Diagnostics.

[11] Qiu X., Yan F., Liu H. (2023). A difference attention ResNet-LSTM network for epileptic seizure detection using EEG signal. Biomed. Signal Process. Control.

[12] Poorani S., Balasubramanie P. (2023). Deep learning based epileptic seizure detection with EEG data. Int. J. Syst. Assur. Eng. Manag..

[13] Varlı M., Yılmaz H. (2023). Multiple classification of EEG signals and epileptic seizure diagnosis with combined deep learning. J. Comput. Sci..

[14] Mir W.A., Anjum M., Shahab S. (2023). Deep-EEG: An optimized and robust framework and method for EEG-based di-agnosis of epileptic seizure. Diagnostics.

[15] Lebal A., Moussaoui A., Rezgui A. (2023). Epilepsy-Net: Attention-based 1D-inception network model for epilepsy detection using one-channel and multi-channel EEG signals. Multimed. Tools Appl..

[16] Ilias L., Askounis D., Psarras J. (2023). Multimodal detection of epilepsy with deep neural networks. Expert Syst. Appl..

[17] McCallan N., Davidson S., Ng K.Y., Biglarbeigi P., Finlay D., Lan B.L., McLaughlin J. (2023). Epileptic multi-seizure type classification using electroencephalogram signals from the Temple University Hospital Seizure Corpus: A review. Expert Syst. Appl..

[18] Ficici C., Telatar Z., Erogul O. (2023). Localization of epileptic focus by gray matter reduction analysis from brain MR images for temporal lobe epilepsy patients. Biomed. Signal Process. Control.

[19] Fearns N., Birk D., Bartkiewicz J., Rémi J., Noachtar S., Vollmar C. (2023). Quantitative analysis of the morphometric analysis program MAP in patients with truly MRI-negative focal epilepsy. Epilepsy Res..

[20] Yin Y., Wang F., Ma Y., Yang J., Li R., Li Y., Liu H. (2023). Structural and functional changes in drug-naïve benign childhood epilepsy with centro-temporal spikes and their associated gene expression profiles. Cereb. Cortex.

[21] Aslam S., Rajeshkannan R., Sandya C.J., Sarma M., Gopinath S., Pillai A. (2022). Statistical asymmetry analysis of volumetric MRI and FDG PET in temporal lobe epilepsy. Epilepsy Behav..

[22] Jber M., Habibabadi J.M., Sharifpour R., Marzbani H., Hassanpour M., Seyfi M., Mobarakeh N.M., Keihani A., Hashemi-Fesharaki S.S., Ay M. (2021). Temporal and extratemporal atrophic manifestation of temporal lobe epilepsy using voxel-based morphometry and corticometry: Clinical application in lateralization of epileptogenic zone. Neurol. Sci..

[23] Li Z., Gao Q., Peng K., Lin J., Wang W., Wang W., Deng B. (2021). Quantitative evaluation of gray matter alterations in patients with mesial temporal lobe epilepsy (MTLE). Neurosci. Inform..

[24] Shigemoto Y., Sato N., Sone D., Maikusa N., Yamao T., Kimura Y., Chiba E., Suzuki F., Fujii H., Takayama Y. (2021). Single-subject gray matter networks in temporal lobe epilepsy patients with hippocampal sclerosis. Epilepsy Res..

[25] Chen S., Zhang J., Ruan X., Deng K., Zhang J., Zou D., He X., Feng L., Bin G., Zeng H. (2020). Voxel-based morphometry analysis and machine learning based classification in pediatric mesial temporal lobe epilepsy with hippocampal sclerosis. Brain Imaging Behav..

[26] Riederer F., Seiger R., Lanzenberger R., Pataraia E., Kasprian G., Michels L., Beiersdorf J., Kollias S., Thomas C., Johannes H. (2020). Voxel-Based Morphometry—From Hype to Hope. A Study on Hippocampal Atrophy in Mesial Temporal Lobe Epilepsy. AJNR Am. J. Neuroradiol..

[27] Uher D., Drenthen G.S., Schijns O.E., Colon A.J., Hofman P.A., van Lanen R.H., Backes W.H. (2023). Advances in Image Processing for Epileptogenic Zone Detection with MRI. Radiology.

[28] Gupta S., Razdan R., Hanumanthu R., Tomycz L., Ghesani N., Pak J., Kannurpatti S.S. (2022). MRI based composite parameter of multiple tissue types for improved patient-level hemispheric and regional level lateralization in pediatric epilepsy. Magn. Reson. Imaging.

[29] Fallahi A., Pooyan M., Habibabadi J.M., Nazem-Zadeh M.R. (2021). Comparison of multimodal findings on epileptogenic side in temporal lobe epilepsy using self-organizing maps. Magn. Reson. Mater. Phys. Biol. Med..

[30] Jing H., Takigawa M., Benasich A.A. (2022). Relationship of nonlinear analysis, MRI and SPECT in the lateralization of temporal lobe epilepsy. Eur. Neurol..

[31] The MathWorks Inc. Deep Learning Toolbox (R2021a). https://www.mathworks.com.

[32] Ficici C. (2022). MRI and EEG Data Correlation for Epilepsy Detection. Ph.D. Thesis.

[33] Chan S., Erickson J.K., Yoon S.S. (1997). Limbic system abnormalities associated with mesial temporal sclerosis: A model of chronic cerebral changes due to seizures. Radiographics.

[34] Ashburner J. (2007). A fast diffeomorphic image registration algorithm. NeuroImage.

[35] Ashburner J., Friston K.J. (2005). Unified segmentation. NeuroImage.

[36] GitHub. https://github.com/otoolej/eeg_viewer.

[37] Beheshti I., Sone D., Maikusa N., Kimura Y., Shigemoto Y., Sato N., Matsuda H. (2021). Accurate lateralization and classification of MRI-negative 18F-FDG-PET-positive. Comput. Biol. Med..

[38] Nazem-Zadeh M.R., Schwalb J.M., Elisevich K.V., Bagher-Ebadian H., Hamidian H., Akhondi-Asl A.R., Jafari-Khouzani K., Soltanian-Zadeh H. (2014). Lateralization of temporal lobe epilepsy using a novel uncertainty analysis of MR diffusion in hippocampus, cingulum, and fornix, and hippocampal volume and FLAIR intensity. J. Neurol. Sci..

[39] Jafari-Khouzani K., Elisevich K., Patel S., Smith B., Soltanian-Zadeh H. (2010). FLAIR signal and texture analysis for lateralizing mesial temporal lobe epilepsy. NeuroImage.

[40] Türk Ö., Özerdem M.S. (2019). Epilepsy detection by using scalogram based convolutional neural network from EEG signals. Brain Sci..

[41] Daoud H., Bayoumi M. (2019). Deep learning approach for epileptic focus localization. IEEE Trans. Biomed. Circuits Syst..

